# Global Trends in Alcohol Control Policies Between 2010–2019

**DOI:** 10.21203/rs.3.rs-8311355/v1

**Published:** 2025-12-23

**Authors:** Juan Pablo Arab, Luis Antonio Díaz, Hyundam Gu, Eduardo Fuentes-López, Francisco Idalsoaga, David Marti-Aguado, Patrick Kamath, ASHWANI SINGAL, Ramon Bataller, Marco Arrese, Leire Agirre-Garrido, Trenton White, Jeffrey Lazarus, Frank Murray

**Affiliations:** Virginia Commonwealth University School of Medicine; MASLD Research Center, Division of Gastroenterology and Hepatology, University of California San Diego; Stravitz-Sanyal Institute for Liver Disease and Metabolic Health, Virginia Commonwealth University School of Medicine; Departamento de Ciencias de la Salud, Facultad de Medicina, Pontificia Universidad Católica de Chile; Departamento de Ciencias de la Salud, Facultad de Medicina, Pontificia Universidad Catolica de Chile; Digestive Disease Department, Clinic University Hospital, INCLIVA Health Research Institute; Mayo Clinic; University of Louisville School of Medicine; Division of Gastroenterology, Hepatology and Nutrition, Pittsburgh Liver Research Center, University of Pittsburgh Medical Center (UPMC), Pittsburgh, PA; Departmento de Gastroenterología, Escuela de Medicina, Pontificia Universidad Católica de Chile; Barcelona Institute for Global Health (ISGlobal), Hospital Clinic, University of Barcelona; Barcelona Institute for Global Health (ISGlobal), Hospital Clínic, University of Barcelona; ISGlobal; Dept of Medicine, Royal College of Surgeons in Ireland, Beaumont Hospital

**Keywords:** ethanol, alcohol use disorder, alcoholic liver disease, alcohol-associated liver disease, cirrhosis, neoplasms

## Abstract

**Background & Aims:**

Alcohol is a leading global risk factor, over 200 diseases and injuries, but policy adoption varies. We updated the Alcohol Preparedness Index (API) to track global and regional policy trends from 2010–2019.

**Methods:**

Using WHO GISAH data for 141 countries, we scored five domains (policy frameworks; production/pricing/taxation; marketing/availability; drink-driving; monitoring/surveillance). The primary endpoint was % change in API. Trends were summarized by WHO region and correlates tested in adjusted regression.

**Results:**

In 2019, <25% of countries had a strong national alcohol plan and 40% had none. Production/pricing/taxation increased most (27%→78%); monitoring/surveillance reached strong levels in 47.5%. Median API increased from 57.0 [39.0–82.0] (2010) to 74.8 [58.3–83.5] (2016) and stayed above baseline in 2019 (67.8 [51.2–83.8]). Larger population predicted less API gain (β=−19.1, p<0.05); HDI and per-capita consumption did not.

**Conclusions:**

Global alcohol policy preparedness increased from 2010–2019.

## INTRODUCTION

Alcohol use remains one of the leading global risk factors for morbidity and mortality, accounting for an estimated 2.6 million deaths annually and contributing substantially to the global burden of disease.^1^ It is a causal factor in many health conditions, most prominently alcohol-associated liver disease (ALD), hepatocellular carcinoma (HCC), six other cancers, cardiovascular disease (CVD) and over 200 illnesses and injuries.^2,3^ Despite its wide-reaching harmful impact, alcohol use is under-regulated globally, with major disparities in both the trends of alcohol consumption and national strategies and their implementation.^4^

Recognizing the global health harms caused by alcohol, the World Health Organization (WHO) launched the Global Strategy to Reduce the Harmful Use of Alcohol in 2010. Subsequently, the SAFER initiative was introduced in 2018 to promote the implementation of evidence-based, high-impact interventions.^5,6^ These include taxation and pricing policies, marketing restrictions, reduced availability, and improved access to treatment. Despite this, implementation of these evidence-based measures has been uneven, particularly in low- and middle-income regions. While many studies have shown that individual alcohol control policies can reduce overall per capita alcohol consumption, fewer have examined their relationship to long-term health consequences, including liver-related outcomes.^7–10^ A recent multinational analysis developed the Alcohol Preparedness Index (API) and demonstrated a strong inverse relationship with mortality from ALD, HCC, cancer, and CVD in the long term.^11^

Building on the prior work, the current study evaluates global and regional API trends over time. By integrating recent sociodemographic data with updated API scores, we also aim to clarify the role and distribution of alcohol-related public health policy (PHP) globally and to provide renewed insights for global health governance and policy planning. Highlighting the national variabilities of API may play as a driver for policy implementation in those countries with low API.

## METHODS

### Study design and participating countries

This study examined the establishment of alcohol-related PHPs by using the API across countries between 2010 and 2019. This updated version of the study expands upon the original analysis, which was based solely on PHP data from 2010, by incorporating additional assessments from 2016 and 2019, updated by the WHO Global Information System of Alcohol and Health at each time point.^11^ Data collection was performed independently by two researchers (Hyundam Gu and Luis Antonio Díaz); any discrepancies were addressed by a third member of the team (Juan Pablo Arab). Missing data in one or two API domains on PHPs for 2019 were imputed with data from 2016, whereas countries with missing data in three or more domains were excluded from the analysis. PHPs were classified using the 2018 WHO policy classification.^12^ Data was also categorized into groups according to WHO regions. Sociodemographic variables were retrieved from the World Bank Open Data repository (http://databank.worldbank.org) and Human Development Reports from the United Nations Development Programme (https://hdr.undp.org/content/human-development-report-2020).

To evaluate the level of alcohol-related PHP implementation across countries, we applied a five-domain scoring instrument originally developed and described in detail in our previous manuscript. Briefly, the instrument was based on policy items available in the WHO GISAH database and organized into five key domains:^11^ 1. National alcohol policy frameworks; 2. Controls on production, pricing, and taxation; 3. Restrictions on marketing and physical availability; 4. Drink-driving countermeasures; and 5. Surveillance and monitoring systems. The instrument structure and scoring criteria were preserved in this updated analysis to ensure comparability over time.^11^ Given the public nature of the datasets and the aggregated level of analysis, an institutional review board was not mandated. This study follows the principles outlined in the GATHER (Guidelines for Accurate and Transparent Health Estimates Reporting) statement.^14^

### Primary and Secondary Outcomes

The primary outcome was to estimate the API in 2016 and 2019. The secondary outcomes include the assessment of trends of API between 2010 and 2019, exploring the regional differences, and identifying indicators of a higher establishment of alcohol-related PHPs over time.

### Statistical analysis

Categorical variables were summarized using frequencies and percentages. We assessed the normality of continuous data using the Shapiro–Wilk test. Continuous variables with a normal distribution were described with their mean and standard deviation, while variables without a normal distribution were summarized using the median and interquartile range [IQR].

We used multiple correspondence analysis (MCA) to estimate the API for each country derived from the scores from the 5-item instrument, based on the previously published methodology.^11^ MCA combines all the information observed in the categorical values for the 5-item instrument into a single factor that acts as a weighted summary of each possible different level indicator combination for each country. The weighted summary has an assigned weight for each individual level of the indicator, which combines to give the full score of the country. We standardized the obtained indicator in order to be able to compare the performance among countries easily. Thus, the API ranged from 0 to 100, 0 being the lowest preparedness and 100 the highest.

To assess the main variables that could explain the changes in API over time, we performed an adjusted linear regression. The covariates included population (log), working-age population, gross domestic product (GDP) per capita (log), Human Development Index (HDI), alcohol per capita consumption (APC), and the World Bank income group. In addition, we included covariates of change of indices of HDI, APC and population from 2010 – 2020. We fit ordinary least squares (OLS) models of increasing complexity and compared parsimony by Akaike’s Information Criterion (AIC). Multicollinearity was assessed with VIFs (and GVIF 1/[2*Df] when factors were present), guiding selection of a prespecified parsimonious model retaining these covariates. Assumptions were checked with standard residual diagnostics; two-sided alpha=0.05 was used for inference. All analyses were conducted using the R statistical package (R software version 4.5.0; R Foundation for Statistical Computing, Vienna, Austria).

## RESULTS

### Baseline Characteristics of Participant Countries

A total of 141 countries (out of 194 countries according to the WHO member countries) were included in the analysis and stratified by the WHO regions. In 2020, 50 countries (36%) were high-income, 39 (28%) upper-middle income, 35 (25%) lower-middle income, and 16 (11%) were low-income. The proportion of high-income countries was greatest in Europe (68.8%), lowest in Africa (3%), and absent in Southeast Asia (0.0%). Across all countries, the median working age proportion of the adult population was 65% [61.3–67.2%]. Economic and health indicators showed substantial heterogeneity across regions. The global median GDP per capita was $6,718 USD, ranging from $1,117 USD in Africa to $23,703 USD in Europe. The HDI showed a similar distribution across the region, with a median value worldwide of 0.77 [0.65–0.88]. The median APC in 2020 was 5.65 liters [2.90–9.33], with Europe reporting the highest consumption of 9.60 [7.10 – 10.80] liters and the Eastern Mediterranean the lowest, 0.10 [0.05 – 0.35] liters. The main baseline characteristics of WHO regions are described in Table 1.

### Establishment of Alcohol-related Public Health Policies Between 2010–2019

Between 2010 and 2016, many countries made small steps forward on national plans, pricing and taxation, and marketing controls (strong levels, from 26% to 29%, from 23% to 26%, and from 27% to 33%, respectively). The number of countries with strong drink-driving policies decreased slightly (from 13% to 11%), and most countries remained in the moderate category (75% in 2010 and 76% in 2016). For monitoring and surveillance, 39% (n = 55) of countries were classified as strong in 2010 but by 2016, no country met the strong criteria, while 84% (n = 118) were classified at the moderate category.

By 2019, fewer than one-quarter (24%) of countries had developed a strong national plan on alcohol, while 40% had no written plan. Drink-driving countermeasures were widely adopted, with nearly three-quarters of countries scoring at the moderate level (74%), although only 8.5% had strong zero-tolerance laws. Control over production, pricing, and taxation had become one of the stronger domains, with approximately 78% of countries reaching a strong level. Monitoring and surveillance systems improved compared with earlier years: nearly half of the countries (48%) achieved a strong system by 2019.

Over time, modest improvements in API were observed. Between 2010 and 2019, strong taxation and pricing measures increased substantially, from 27% in 2010 to 78% in 2019. Monitoring and surveillance showed gains, whereas progress in drink-driving regulation and marketing controls was not significant, with most countries plateauing at the moderate level. In 2019, the European region, along with several countries in Southeast Asia and the Western Pacific, had higher proportions of strong or moderate policies across multiple items, whereas the Eastern Mediterranean and African regions more often remained in the low category for key policy areas (**Supplementary figure 1**).

**Figure 2:**
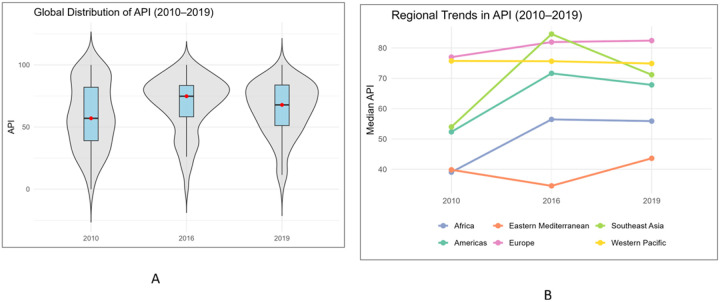
Global and regional trends in the Alcohol Preparedness Index (API), 2010–2019 **Figure 2A.** Global distribution of API, 2010, 2016, and 2019. **Figure 2B.** Regional trends by WHO region, 2010, 2016, and 2019.

The global distribution of the API improved between 2010 and 2019 (Figure 2). In 2010, the median API across 141 countries was 57.0 [39.0–82.0]. This increased to 74.8 [58.3–83.5] in 2016 and 67.8 [51.2–83.8] in 2019 (Figure 2A). The 10 countries with the steepest relative gains between 2010 and 2019 were Antigua and Barbuda, Burundi, Chad, Paraguay, Barbados, Dominica, Guatemala, Bangladesh, Belize, and Cabo Verde (**Supplementary figure 3**). The 10 countries with the highest API were also described in **Supplementary Figure 4**.

In 2019, the median API varied widely by WHO region, ranging from 43.6 [IQR 27.8–54.4] in the Eastern Mediterranean to 82.4 [IQR 71.1 – 85.3] in Europe. Western Pacific and Southeast Asia also showed high central values, 74.9 [IQR 55.9–83.8] and 71.2 [IQR 54.2–90.0], respectively. The Americas reached 67.8 [IQR 54.3–75.7] and Africa sat in the mid-range at 55.9 [IQR 41.5–64.5]. Regional trends in the API are shown in Figure 2B. Europe and the Western Pacific showed steady improvements over the decade, while the Americas remained relatively stable. Africa experienced a marked rise between 2010 and 2016, and the Eastern Mediterranean showed little change across the study period.

### Main predictors of a Higher API by 2019

The regression analyses examined how demographic and economic characteristics measured in 2020 relate to changes in API from 2010 to 2019. In the final model, population size was a strong, highly significant negative correlation of API change (β = −19.1, p < 0.05), whereas HDI and APC were not statistically significant (Table 2).

Given the relative stability of population size and development indicators across the decade, the findings demonstrate that countries with larger 2020 populations tended to experience smaller prior changes in API, while other demographic and economic variables added little explanatory power.

Supplementary table 5 lists the ten countries with the lowest AUD prevalence (%) and the lowest rates of HCC incidence, alcohol-attributable HCC incidence, HCC mortality, cirrhosis mortality, and cardiovascular mortality in 2020. Countries highlighted in the table (Belize, Burundi, Argentina) are those that also ranked among the top 10 for either the largest increase in API score during the time or the highest API scores in 2019. The table indicates which countries simultaneously exhibit low levels of alcohol-related health indicators and high or improving API performance.

## DISCUSSION

In this global study, we assembled a cross-national dataset for 141 countries, scored alcohol policy strength across WHO domains to estimate the API at three time points (2010, 2016, and 2019), characterized regional patterns, and examined socioeconomic contexts associated with changes in API over the decade.

A key finding is that the implementation of alcohol-related public health policies was uneven across domains and regions, with pricing/taxation and monitoring/surveillance showing the clearest strengthening. The API rose globally but with marked regional differences: Europe and the Western Pacific generally improved, the Americas were relatively stable, Africa experienced a rise, and the Eastern Mediterranean changed little. Countries with larger populations tended to exhibit smaller gains in API over the period, whereas other demographic and economic characteristics offered limited additional explanatory value.

Historically, several indices have been developed to assess alcohol policy; however, most capture only a subset of evidence-based measures. For example, the International Alcohol Control (IAC) Policy Index and the International Alcohol Policy and Injury Index (APII) demonstrate correlations with alcohol intake and alcohol-related outcomes, yet they do not encompass major components of implemented policy packages.^9,10^, We have previously demonstrated that higher API values were associated with lower ALD mortality, lower AUD prevalence, and lower alcohol-attributable HCC prevalence, as well as reduced cancer and cardiovascular mortality.^11^ The API is also, to our knowledge, the first index to include policies that facilitate access to screening, brief interventions, and treatment, core elements of the WHO SAFER recommendations.

From policy development and implementation perspectives, our findings point to practical directions for a more comprehensive future index. Controls on production, pricing, and taxation have improved in many countries, consistent with extensive evidence linking higher prices to lower consumption and harm, and with WHO “best buys” guidance that assists governments in prioritizing evidence based, feasible to implement, and cost-effective actions.^15,16^ Recent WHO “quick buys” further emphasize measures likely to yield more immediate impact (within 5 years).^17^ Incorporating urgency and expected time-to-impact into the future index could be achieved by weighting policy items accordingly (e.g., higher weights for interventions identified as rapidly effective, with explicit documentation of the weighting scheme, or greater weighting for greater price/affordability measures and advertising restrictions) to reflect both effectiveness and timeliness in a single score. Minimum Unit Pricing (MUP) is another pricing measure increasingly adopted in several jurisdictions.^18^ Following implementation in Scotland and parts of Europe beginning in 2018, emerging evidence indicates reductions in consumption and in alcohol-related hospitalizations and deaths, with reduction in health inequity.^19^ Because these initiatives are relatively recent (particularly in high-income settings), MUP was not incorporated into the present API spanning the prior decade. Beyond pricing policies, additional elements remain difficult to capture at scale, including potential restrictions on digital and social media marketing (which impact youth drinking) and the presence and prominence of warning labels on containers.^20^

Several factors were not included in our analyses, such as ethnicity, the distribution of comorbidities, and educational attainment, and substantial cross-country variation in how policies are implemented in practice. Global, systematic data on implementation quality remain limited, especially for the period before and after WHO recommendations, even though country-level studies link specific policies to health outcomes.^21–23^ Alcohol policy is typically dispersed across multiple government sectors, making evaluation and enforcement tracking challenging.^12^ In parallel, the alcohol industry has increasingly engaged in influencing policy in many countries, including through corporate “social” responsibility initiatives and highly funded and successful lobbying, potentially shaping the adoption and strength of measures.^24–26^ The extent to which variation in implementation and industry engagement influences alcohol preparedness and how these factors interact with consumption and harm remains insufficiently researched.

In conclusion, we introduce a standardized alcohol preparedness index that translates national alcohol policies into a comparable metric and apply it to 141 countries for a decade. The index shows uneven progress across domains and regions; however, the overall global trajectory is upward. Looking ahead, we will evolve the index to better reflect real-world implementation over time, incorporate emerging policy approaches as evidence and data grow, and strengthen its utility for tracking progress and informing public health decisions.

## Supplementary Material

Supplementary Files

This is a list of supplementary files associated with this preprint. Click to download.

- Appendix.pdf

## Figures and Tables

**Figure 1:**
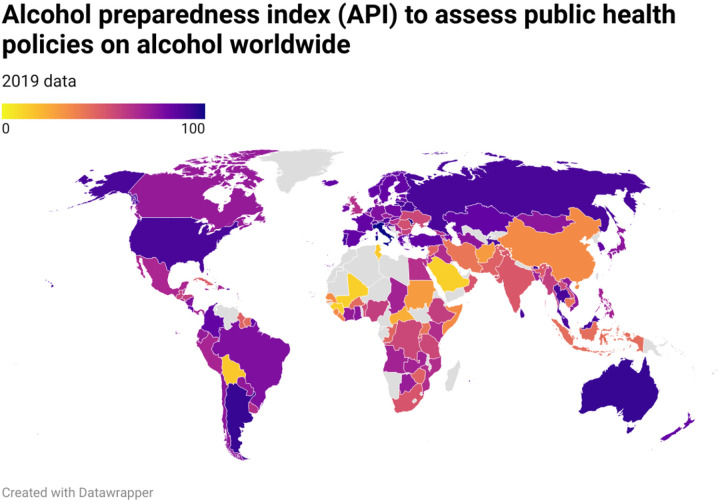
Alcohol preparedness index (API) in 2019. Data from the World Health Organization Global Information System on Alcohol and Health in 2019 was used. Missing data was imputed by using the data available in 2016 for each country. The API was estimated based on the previously published 5-instrument using multiple correspondence analysis. API ranges from 0 to 100, 0 being the lowest establishment of alcohol-related public health policies and 100 the highest.

**Table 1.** Baseline characteristics of the 141 included countries by the World Health Organization (WHO) region.

		Global	Africa	Americas	Eastern Mediterranean	Europe	Southeast Asia	Western Pacific
Countries by WHO region		141	29	32	12	48	7	13
Income level, number of included countries	High	50 (35.7)	1 (3.4)	8 (25.0)	3 (25.0)	33 (68.8)	0 (0.0)	5 (41.7)
	Upper-middle	39 (27.9)	2 (6.9)	19 (59.4)	2 (16.7)	13 (27.1)	1 (14.3)	2 (16.7)
	Lower-middle	35 (25.0)	13 (44.8)	5 (15.6)	4 (33.3)	2 (4.2)	6 (85.7)	5 (41.7)
	Low	16 (11.4)	13 (44.8)	0 (0.0)	3 (25.0)	0 (0.0)	0 (0.0)	0 (0.0)
Total population, median (2020)		11,538,604	17,224,679	6,603,739	35,310,745	7,794,040	71,641,484	29,769,403
Percentage of 15 – 64 years old		64.91 [61.26, 67.23]	54.86 [52.71, 57.05]	66.53 [65.41, 68.96]	61.35 [57.48, 67.23]	64.96 [63.70, 66.67]	67.56 [66.59, 69.48]	65.23 [63.98, 69.34]
Population of 15 – 64 years old, median (2020)		7,154,627	9,425,714	4,251,397	21,789,196	5,018,358	50,818,801	20,094,147
Gross domestic product (GDP) (2020)		6717.65 [2235.50, 20896.34]	1117.42 [845.77, 2019.66]	8089.48 [5311.56, 13158.75]	3249.95 [576.44, 4090.33]	23703.44 [9776.62, 46274.66]	3191.67 [2077.95, 3850.65]	10292.50 [3457.42, 40484.28]
Alcohol per capita consumption (APC) (2020)		5.65 [2.90, 9.33]	3.80 [2.70, 6.00]	5.20 [3.68, 6.85]	0.10 [0.05, 0.35]	9.60 [7.10, 10.80]	1.90 [0.15, 3.50]	7.20 [5.43, 8.50]
Human development index (HDI) (2020)		0.77 [0.65, 0.88]	0.55 [0.49, 0.61]	0.77 [0.73, 0.81]	0.73 [0.55, 0.76]	0.90 [0.83, 0.93]	0.69 [0.66, 0.74]	0.81 [0.75, 0.93]

* All sociodemographic factors showed *p< 0.001*

** Categorical variables; n (%)

*** Continuous variables; median [IQR]

**Table 2.** Predictors of change (%) of API.

Independent variable	Coefficient
Population (log) (2010)	−19.050 (4.482)*
APC (2010)	−1.758 (2.641)
HDI (2010)	−79.266 (69.541)
Population change (log) between 2010–2020	−3.398 (3.309)
APC change between 2010–2020	−0.567 (8.531)
HDI change between 2010–2020	−58.496 (70.163)
	
Constant 422.214	
	
R = 0.399	
F-ratio = 3.654*	

* p < 0.05

**Note:** Coefficients are unstandardized OLS partial regression slopes; standard errors in parentheses. Residual standard error = 92.14; Adjusted R^2^ = 0.116.

## References

[1] Global status report on alcohol and health and treatment of substance use disorders. https://www.who.int/publications/i/item/9789240096745 (2024).

[2] Díaz L. A., Arab J. P., Louvet A., Bataller R. & Arrese M. The intersection between alcohol-related liver disease and nonalcoholic fatty liver disease. Nat. Rev. Gastroenterol. Hepatol. 20, 764–783 (2023). 37582985 10.1038/s41575-023-00822-y

[3] Meza V. Alcohol consumption: Medical implications, the liver and beyond. Alcohol Alcohol 57, 283–291 (2022). 35333295 10.1093/alcalc/agac013

[4] Nishtar S. Time to deliver: report of the WHO Independent High-Level Commission on NCDs. Lancet 392, 245–252 (2018). 29866374 10.1016/S0140-6736(18)31258-3

[5] World Health Organization. Global Strategy to Reduce the Harmful Use of Alcohol. (World Health Organization, Genève, Switzerland, 2010). 10.2471/BLT.19.241737 PMC7047030 32132758

[6] Rekve D. Prioritising action on alcohol for health and development. BMJ 367, l6162 (2019). 31810905 10.1136/bmj.l6162

[7] Madureira-Lima J. & Galea S. Alcohol control policies and alcohol consumption: an international comparison of 167 countries. J. Epidemiol. Community Health 72, 54–60 (2017). 29061844 10.1136/jech-2017-209350

[8] Brand D. A., Saisana M., Rynn L. A., Pennoni F. & Lowenfels A. B. Comparative analysis of alcohol control policies in 30 countries. PLoS Med. 4, e151 (2007). 17455992 10.1371/journal.pmed.0040151 PMC1876414

[9] PLOS Global Public Health Staff. Correction: Benchmarking alcohol policy based on stringency and impact: The International Alcohol Control (IAC) Policy Index. PLOS Glob. Public Health 2, e0000592 (2022). 36962409 10.1371/journal.pgph.0000592 PMC10021904

[10] Korcha R. A. Development of the international alcohol policy and injury index. Rev. Panam. Salud Publica 42, (2018). 10.26633/RPSP.2018.6 PMC6007029 29937675

[11] Díaz L. A. Association between public health policies on alcohol and worldwide cancer, liver disease and cardiovascular disease outcomes. J. Hepatol. 80, 409–418 (2024). 37992972 10.1016/j.jhep.2023.11.006

[12] World Health Organization. Global Status Report on Alcohol and Health 2018. (World Health Organization, Genève, Switzerland, 2019).

[13] GBD 2019 Diseases and Injuries Collaborators. Global burden of 369 diseases and injuries in 204 countries and territories, 1990–2019: a systematic analysis for the Global Burden of Disease Study 2019. Lancet 396, 1204–1222 (2020). 33069326 10.1016/S0140-6736(20)30925-9 PMC7567026

[14] Stevens G. A. Correction: Guidelines for accurate and transparent health estimates reporting: The GATHER statement. PLoS Med. 13, e1002116 (2016). 27504831 10.1371/journal.pmed.1002116 PMC4978408

[15] Banatvala N., Bovet P., Isaranuwatchai W. & Bertram M. Y. Best buys and other recommended interventions for NCD prevention and control. in Noncommunicable Diseases 246–252 (Routledge, London, 2023). doi:10.4324/9781003306689-38.

[16] Elder R. W. The effectiveness of tax policy interventions for reducing excessive alcohol consumption and related harms. Am. J. Prev. Med. 38, 217–229 (2010). 20117579 10.1016/j.amepre.2009.11.005 PMC3735171

[17] Galea G. Quick buys for prevention and control of noncommunicable diseases. Lancet Reg. Health Eur. 52, 101281 (2025). 40452915 10.1016/j.lanepe.2025.101281 PMC12126618

[18] Anderson P., Stockwell T., Natera G. & Kaner E. Minimum unit pricing for alcohol saves lives, so why is it not implemented more widely? BMJ 384, e077550 (2024). 38471733 10.1136/bmj-2023-077550

[19] Maharaj T. Impact of minimum unit pricing on alcohol-related hospital outcomes: systematic review. BMJ Open 13, e065220 (2023). 10.1136/bmjopen-2022-065220 PMC9900069 36737089

[20] Empowering public health advocates to navigate alcohol policy challenges: alcohol policy playbook. https://www.who.int/europe/publications/i/item/WHO-EURO-2024-5624-45389-76520 (2024).

[21] Herttua K., Mäkelä P. & Martikainen P. Changes in alcohol-related mortality and its socioeconomic differences after a large reduction in alcohol prices: a natural experiment based on register data. Am. J. Epidemiol. 168, 1110–8; discussion 1126–31 (2008). 18718894 10.1093/aje/kwn216 PMC2727242

[22] Wagenaar A. C., Tobler A. L. & Komro K. A. Effects of alcohol tax and price policies on morbidity and mortality: a systematic review. Am. J. Public Health 100, 2270–2278 (2010). 20864710 10.2105/AJPH.2009.186007 PMC2951962

[23] Zhao J. & Stockwell T. The impacts of minimum alcohol pricing on alcohol attributable morbidity in regions of British Colombia, Canada with low, medium and high mean family income. Addiction 112, 1942–1951 (2017). 28600882 10.1111/add.13902

[24] McCambridge J., Kypri K., Sheldon T. A., Madden M. & Babor T. F. Advancing public health policy making through research on the political strategies of alcohol industry actors. J. Public Health (Oxf.) 42, 262–269 (2020). 31220307 10.1093/pubmed/fdz031 PMC7297281

[25] Maani N., van Schalkwyk M. C. & Petticrew M. The perils of partnership: Interactions between Public Health England, Drinkaware, and the Portman Group surrounding the Drink Free Days campaign. Int. J. Health Policy Manag. 13, 8245 (2024). 39099521 10.34172/ijhpm.2024.8245 PMC11607520

[26] Mialon M. & McCambridge J. Alcohol industry corporate social responsibility initiatives and harmful drinking: a systematic review. Eur. J. Public Health 28, 664–673 (2018). 29697783 10.1093/eurpub/cky065 PMC6051456

